# Lacosamide on background eeg activity in brain tumor‐related epilepsy patients: A case series study

**DOI:** 10.1002/brb3.1067

**Published:** 2018-10-17

**Authors:** Marta Maschio, Alessia Zarabla, Andrea Maialetti, Francesca Sperati, Loredana Dinapoli, Sabrina Dispenza, Gianluca Petreri, Tonino Cantelmi

**Affiliations:** ^1^ Center for Tumor‐related Epilepsy, UOSD Neurology Regina Elena National Cancer Institute Rome Italy; ^2^ Biostatistics/Scientific Direction Regina Elena National Cancer Institute Rome Italy; ^3^ Service of Psychiatry Regina Elena National Cancer Institute Rome Italy

**Keywords:** antiepileptic drugs, brain tumor‐related epilepsy, EEG background activity, efficacy, lacosamide, quantitative EEG

## Abstract

**Objective:**

Therapeutic doses of antiepileptic drugs (AEDs) may alter EEG background activity, which is considered an index of the functional state of the brain. Quantitative analysis (qEEG) of EEG background activity is a valid instrument to assess the effects of many centrally active drugs on the central nervous system, including AEDs. Lacosamide (LCM) is a new AED that could be a valid therapeutic choice in patients with brain tumor‐related epilepsy (BTRE).

**Methods:**

We used qEEG to analyze the possible effect of LCM as an add‐on, on background EEG activity after 4 months in patients with BTRE.

**Results:**

We consecutively recruited sixteen patients with BTRE: Five dropped out for disease progression, five for scarce compliance, and six completed the study. For these reasons qEEG was performed at first visit and after 4 months only in six patients. For all frequency bands, LCM revealed no changes of mean relative power during rest with eyes closed, hyperpnoea (HP), and mental arithmetic task (MA); significant increment was found only in the theta mean relative power during opening and closing eyes (BR). After four months of therapy with LCM, one patient was seizure free, four had a seizure reduction ≥50%, and one showed a worsening in seizure frequency <50%.

**Conclusion:**

Despite the limitation of a small series, these findings suggest that LCM seems to have only a mild interference on EEG background activity and confirm that LCM has a good efficacy on seizure control in patients with BTRE. This is the first study that evaluates the effect of LCM on background EEG activity, using qEEG in BTRE patients. Future research in this area could include prospective studies with qEEG for a longer follow‐up period to assess the impact of AEDs on brain functions in this particular fragile patient population.

## 1 INTRODUCTION

A multilead computer‐assisted quantitative analysis of human scalp‐recorded EEG (qEEG) is a simple and objective instrument to assess the effect of centrally active drugs on the central nervous system (CNS) (Cho et al., 2012); it enables the evaluation of the effects of a drug on regional electrical brain activity, named background EEG activity, considered an index of the functional state of the brain (Cho et al., 2012; Knott, 2000).

The therapeutic doses of antiepileptic drugs (AEDs) may alter EEG background activity; in particular, the conventional AEDs are associated with significant EEG slowing, widely considered an indicator of CNS dysfunction (Cho et al., 2012).

Among new AED, lacosamide (LCM) is a novel well‐tolerated AED without significant pharmacokinetic interactions that is licensed for adjunctive therapy of partial or secondary generalized seizures (Kellinghaus, 2009). It could be a valid therapeutic choice in patients with brain tumor‐related epilepsy (BTRE), often refractory to AEDs (Maschio & Dinapoli, 2012).

While there are studies in the literature on the efficacy of LCM in seizure control in BTRE patients (Maschio et al., 2011; Saria et al., 2013), there have been no studies to date on the effect of LCM on EEG background activity in this patient population.

For this reason, we analyze the possible effect of LCM, as an add‐on, on EEG background activity, using qEEG, in adult BTRE patients with uncontrolled partial‐onset seizures.

## 2 MATERIALS AND METHODS

### 2.1 Patients

We consecutively recruited sixteen patients with BTRE (twelve male and four female, mean age 44.1 years), who have had at least one seizure in the month preceding recruitment, despite having taken AEDs at highest dose tolerable.

Fourteen patients had high‐grade glioma (HGG), two had low‐grade glioma (LGG). Twelve patients were in chemotherapy (10 with temozolomide and two with lomustine). One patient had simple partial seizures, two had complex partial seizures, five had complex partial seizures secondarily generalized and eight with simple partial seizures secondarily generalized. Four patients were in monotherapy with levetiracetam, one with lamotrigine, one with carbamazepine, one with phenytoin, one with valproic acid, and 8 were in polytherapy.

Epilepsy was diagnosed following guidelines of the International League Against Epilepsy http://www.ilae.org/.

All patients were treated with the current standard care of brain tumor patients, and brain MRI follow‐up was done at the beginning and end of study period. Patients with organic or psychiatric disorders who used drugs interfering with CNS (other than AEDs) were excluded. At first visit, patients underwent a physical and neurological examination including Karnofsky Performance Status (Karnofsky et al., 1951) and Barthel Index (Mahoney & Barthel, 1965) as an index of functional independence in personal and domestic activities of daily living and received a seizure diary. LCM was titrated according to technical file, as first to fifth add‐on therapy, at dosage variable from 200 to 400 mg/day depending on seizure control and the onset of adverse events. The starting dosage was 100 mg/day with a weekly increase of 100 mg/day. The remaining AED therapies were left unmodified. At final follow‐up at 4 months, patients were given complete physical and neurological examination, check on seizure frequency, and active check of adverse events.

The quantitative EEG was to be performed at first visit and after four months of therapy, with the exclusion of patients with disease progression. The presence and severity of LCM side effects were evaluated using Common Terminology Criteria for Adverse Events http://ctep.cancer.gov/protocolDevelopment/electronic_applications/docs/ctcaev4.pdf.

The study was approved by the Institute's Ethical Committee and each participant signed informed consent.

### 2.2 EEG procedures

The EEG machine used was MICROMEDIA BQ2400 Studio ACQDV to 25 channels. Nineteen scalp‐electrodes were placed according to 10–20 International System; electrocardiogram was recorded via additional skin surface electrodes.

Electrode impedance was maintained below 20 Kohm. Filters were set at 1.6 and 70 Hz and the signal was notch filtered. All EEG recordings were acquired with a 256 bit‐sampling rate. Recording sessions included: 10 min at rest with eyes closed (REST), 5 min during hyperpnoea (HP), 5 min during opening and closing eyes (BR), 5 min during mental arithmetic task (MA), of continuous subtraction of same digit from an initial starting number.

The off‐line spectral analysis was performed using Fast Fourier Transform on 5–10 min of EEG signal, manually segmented into >2 s epochs, after visual elimination of interictal epileptiform activity and artifacts. These epochs were collected for each frequency band: alpha (8–12.5) Hz; theta (4–7) Hz; delta (1–3.5) Hz; and beta (13–30) Hz. The relative power of these four bands was computed for 19 monopolar derivations, as the ratio of the absolute power within a given band to the total power of the total frequency range. Relative power values were considered due to their lower intersubject variability (Placidi et al., 2004).

### 2.3 Statistical analysis

We reported continuous data as means and standard deviations. We compared patients pre‐LCM and post‐LCM treatments, and to take into account the small size of our sample, we applied the Wilcoxon nonparametric test to compare median values of electrode frequencies reported in EEG at REST and during MA, HP, and BR. All statistical analyses were carried out with SPSS statistical software version 20 (SPSS Inc., Chicago, IL, USA).

## 3 RESULTS

Five patients dropped out for disease progression, and five patients dropped out for scarce compliance, immediately after enrollment; therefore, six patients completed the study (Table 1). Only six patients could be evaluated by qEEG after 4 months of therapy.

**Table 1:** Patients' clinical and vital data

	Age	Sex	Histology	Site	CT^a^	Seizure type	Previous AED therapy (mg/die)	LCM mg/die	Side effects	Mean number of seizures/month		Scores at baseline	Scores at 4 months
										1 month prior to LCM therapy	After 4 months	KPS/BI	KPS/BI
1	47	F	AA	T left	TMZ	CP + SGTC	LEV 3000 LTG 400 TPM 400	400	None	1	0	100	100
2	40	M	LGO	F left	None	CP + SGTC	LEV 3000 ZNS 200 TPM 100	400	None	30	2	100	100
3	41	M	GBM	P‐O right	TMZ	CP + SGTC	LEV 3000	400	None	2	1	100	100
4	35	F	AOA	P‐right	TMZ	SP + SGTC	LEV 3000 PGB 300	400	None	1	0.25	100	100
5	42	M	AOA	F left	CCNU	SP + SGTC	VPA 1000 LTG 300 LEV 3000 CNZ 1.5	400	None	1	3	100	100
6	49	M	GBM	T left	TMZ	CP + SGTC	LEV 3000	400	None	30	2	100	100

*Notes*

Histological diagnosis: AA, anaplastic astrocytoma; LGO, low‐grade oligodendroglioma; GBM, glioblastoma multiforme; AOA, anaplastic oligoastrocytoma; CT, chemotherapy; CCNU, lomustine, TMZ, temozolomide; seizure type, SP, simple partial, CP, complex partial; SGTC, secondarily generalized tonic‐clonic; AEDs, antiepileptic drugs; CNZ, clonazepam; LEV, levetiracetam; LTG, lamotrigine; PGB, pregabalin; TPM, topiramate; ZNS, zonisamide.

a During follow‐up.

These six patients had all reached the LCM dosage of 400 mg/day. No patients reported side effects due to LCM. In these patients, the oncological disease remained stable. After four months of treatment, no significant changes in functional independence or everyday life activities were found (Table 1) and neuro‐radiological examination (BrainMRI) remained stable.

**Figure 1:**
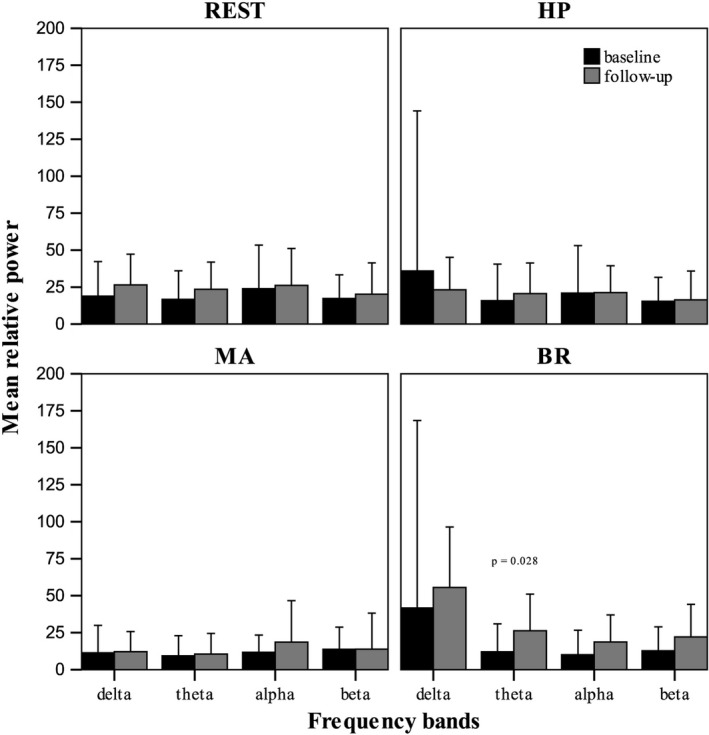
Mean relative power+2 standard deviations (SD) for REST, MA task, HP task and RA task, according to the frequency bands at baseline and follow‐up

Comparison of EEG background activity recorded at REST and during HP, MA, BR, before and after four months of treatment with LCM revealed: 1) no significant changes of mean relative power of any of the frequency bands, in all electrodes, during REST, HP and MA; 2) a significant increment only in the theta mean relative power during BR at 4 months of follow‐up (Table 2; Figure 1).

**Table 2:** Comparison of EEG background activity recorded at rest with eyes closed and during hyperpnoea, mental arithmetic task, opening and closing eyes, before and after four months of treatment with lacosamide

	REST^a^		HP^b^		MA^c^		BR^d^	
	Pre‐LCM^e^	Post‐LCM	Pre‐LCM	Post‐LCM	Pre‐LCM	Post‐LCM	Pre‐LCM	Post‐LCM
Alpha	24.0 ± 29.2	26.2 ± 24.8 n.s.	21.0 ± 32.0	21.3 ± 18.2 n.s.	11.8 ± 11.7	18.7 ± 28.0 n.s.	10.1 ± 8.2	18.8 ± 9.1 n.s.
Theta	16.6 ± 19.4	23.5 ± 18.2 n.s.	15.8 ± 24.7	20.6 ± 20.6 n.s.	9.4 ± 13.6	10.6 ± 13.9 n.s.	12.1 ± 9.4	26.3 ± 12.4 *p* = 0.028
Delta	18.9 ± 23.2	26.4 ± 20.8 n.s.	35.9 ± 108.2	23.2 ± 22.0 n.s.	11.4 ± 18.5	12.1 ± 13.7 n.s.	41.7 ± 63.3	55.5 ± 20.4 n.s.
Beta	17.3 ± 15.8	20.2 ± 21.0 n.s.	15.4 ± 16.2	16.4 ± 19.4 n.s.	13.7 ± 15.1	13.8 ± 24.4 n.s.	12.8 ± 8	22.2 ± 11 n.s.

a REST: Eyes closed.

b HP: Hyperpnoea.

c MA: Mental Arithmetic Tasks.

d BR: Opening and closing eyes.

e LCM: Lacosamide.

The mean seizure number in the month prior to administration of LCM was 27.3; at 4 months, it was 2.9.

After four months of therapy, one patient was seizure free, four had a seizure reduction ≥50%, and one showed a worsening in seizure frequency <50% (Table 1).

## 4 DISCUSSION

In the literature, there have been published several studies regarding the effect of new‐generation AEDs, such as lacosamide, lamotrigine, levetiracetam, and oxcarbazepine, on EEG background activity, using qEEG; however, none of these was conducted on BTRE patients (Cho et al., 2012; Clemens et al., 2006; Giorgi et al., 2013; Meador et al., 2016). Patients with BTRE are a particular population because they have two serious diseases simultaneously and must take multiple therapies (such as chemotherapy and radiotherapy) in addition to AEDs.

BTRE is a rare disease. Although rare, BTRE constitutes 6%–10% of all cases of epilepsy as a whole and 12% of acquired epilepsy (Singh, Rees, & Sander, 2007). Overall, the incidence of epilepsy in BTs, regardless of histological type and anatomical site of the lesion, varies from 35% to 70% (Thom, Blumcke, & Aronica, 2012; You, Sha, & Jiang, 2012). Despite being rare, individuals with this pathology represent an enormous socio‐economic burden to the national healthcare system.

Due to the facts that BTRE is a rare disease, study on this pathology is burden by recruitment difficulties and other challenges common to rare diseases. Among these the most important are: sample size, time (considering the necessity to balance the needs of study participants with the need to publish significant results) and potential high dropout rate/noncompliance due to psychological and physical challenges. Furthermore, patients with brain tumor often do not have the stamina for taking numerous exams, and caregivers have difficulties bringing them to numerous appointments. In this context, our aim was not the evaluation of an LCM on seizure control (i.e., clinical focus) but rather the evaluation of the impact of an AED on background EEG activity. For this reason, patients with disease progression were automatically excluded from the study; in a study with a clinical focus, they would have been allowed to complete final follow‐up.

All drugs that could modify functions of CNS can lead to changes in EEG frequency that can be detected using qEEG (Saletu, Anderer, Saletu‐ Zyhlarz, Arnold, & Pascual‐Marqui, 2002). Therefore, in patients with BTRE, a simple, quickly, and not expensive method such as qEEG could be useful in recognizing subtle CNS dysfunctions that can often remain undetected.

To date, there has been no study in the literature that evaluates the possible effect of LCM on EEG background activity using qEEG in BTRE patients.

In our study, LCM did not induce significant changes on EEG background activity for any of the frequency bands during REST, MA, and HP, with the exception of the theta band activity only during BR that was significantly increased.

Our results are in line with literature data regarding the effect of LCM on EEG background activity in nononcological patients, where it is demonstrated that LCM does not change the background activity (Giorgi et al., 2013). The only modification we observed (theta band activity increased during BR) is consistent with data obtained from a recent randomized double‐blind study (Meador et al., 2016) on EEG effects of LCM in a healthy subject.

We know that the small population and the short follow‐up (4 months) are important limitations of our study that prevents us to draw definitive conclusions; this applies for new studies with a wide and homogeneous sample and a longer follow‐up. Nevertheless, the high dropout rate (due to disease progression and scarce compliance) revealed by our study can be useful information for planning future studies that investigate AEDs and possible CNS involvement in this patient population, using qEEG, making researchers aware of potential difficulties in recruiting these patients.

Although our study had duration of only four months, LCM demonstrated good seizure control, in line with the data in the literature in this patient population (Maschio et al., 2011; Saria et al., 2013).

In conclusion, this preliminary study could be the starting point for future researches in this area using qEEG for a longer follow‐up period, eventually with the neuropsychological test (Cho et al., 2012; Clemens et al., 2006), to assess the impact of AEDs on brain functions in this particular fragile patient population.

## CONFLICT OF INTEREST

The corresponding author declares to have a financial relationship with UCB PHARMA and EISAI s.r.l. (research support). The co‐authors declare that they have no competing interests.
